# Magnetoelectrics: Three Centuries of Research Heading Towards the 4.0 Industrial Revolution

**DOI:** 10.3390/ma13184033

**Published:** 2020-09-11

**Authors:** Nélson Pereira, Ana Catarina Lima, Senentxu Lanceros-Mendez, Pedro Martins

**Affiliations:** 1 Centro/Departamento de Física, Universidade do Minho, 4710-057 Braga, Portugal; nmmsp.18@gmail.com (N.P.); anacatari18@gmail.com (A.C.L.); 2 Algoritmi Center, Minho University, 4800-058 Guimarães, Portugal; 3 INL—International Iberian Nanotechnology Laboratory, 4715-330 Braga, Portugal; 4 BCMaterials, Basque Center for Materials, Applications and Nanostructures, Universidad del País Vasco/Euskal Herriko Unibertsitatea, Science Park, 48940 Leioa, Spain; 5 Basque Foundation for Science (Ikerbasque), 48013 Bilbao, Spain; 6 IB-S Institute of Science and Innovation for Bio-sustainability, Universidade do Minho, 4710-057 Braga, Portugal

**Keywords:** magnetoelectric, piezoelectric, magnetostrictive, multiferroic, IoT, 4.0 industrial revolution

## Abstract

Magnetoelectric (ME) materials composed of magnetostrictive and piezoelectric phases have been the subject of decades of research due to their versatility and unique capability to couple the magnetic and electric properties of the matter. While these materials are often studied from a fundamental point of view, the 4.0 revolution (automation of traditional manufacturing and industrial practices, using modern smart technology) and the Internet of Things (IoT) context allows the perfect conditions for this type of materials being effectively/finally implemented in a variety of advanced applications. This review starts in the era of Rontgen and Curie and ends up in the present day, highlighting challenges/directions for the time to come. The main materials, configurations, ME coefficients, and processing techniques are reported.

## 1. Introduction

**Figure 1:**
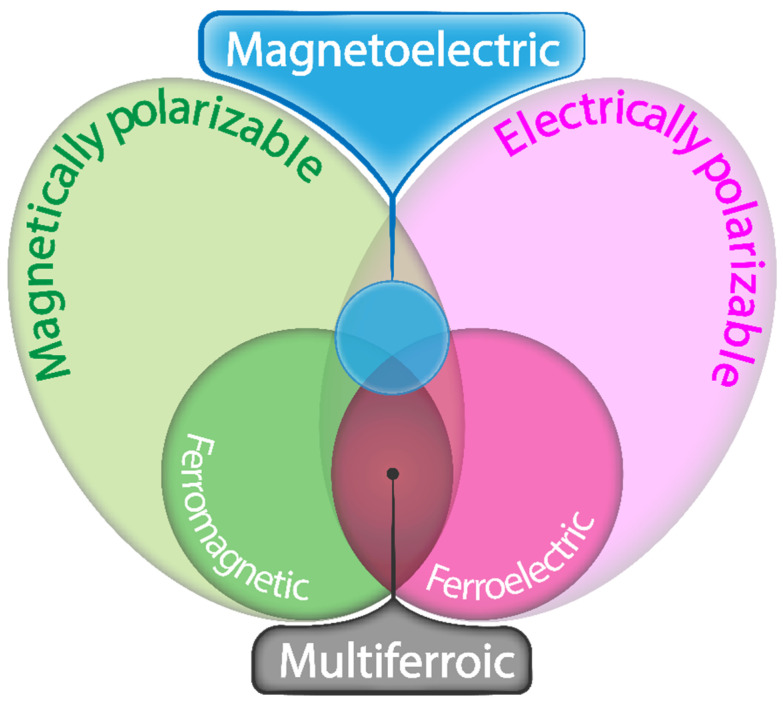
The relation between multiferroic and ME materials, adapted from [1].

Multiferroic materials are formed by at least two ferroic orders: a ferroelectric order where spontaneous and stable polarization can be switched hysteretically by an applied electric field; a ferromagnetic order where spontaneous and stable magnetization can be switched hysteretically by an applied magnetic field or ferroelastic order where spontaneous and stable deformation can be switched hysteretically by an applied stress [1]. The interaction between different ferroic orders can produce effects such as the magnetoelectric (ME) one schematically represented in Figure 1.

Such a ME response is reflected in the appearance of an electric polarization variation when applying an external magnetic field, or in the induced magnetization variation under an external electric field [2]. The initial studies regarding the ME effect by Rontgen in 1888 [3] and Curie in 1894 [4] reported that a moving dielectric became magnetized when placed in a magnetic field and the possibility of intrinsic linear ME behaviour of crystals based on symmetry considerations, respectively. After those considerations, the ME concept started to get increasing attention. The term “magnetoelectric” was introduced by Debye in 1926, after the first failed attempts to experimentally demonstrate the ME effect. Three decades later, in 1959, Dzyaloshinkii reported two evidences of the ME effect: (i) that an electric field induced magnetization on Cr_2_O_3_ and (ii) a magnetic field induced polarization in the same Cr_2_O_3_ [5,6].

After such observations of the ME effect in Cr_2_O_3_, interest in the ME coupling effect declined from some years due to the fact that the low magnitude of ME coupling was not enough to allow ME phase control in devices such as memories or switching devices with magnetic control of the electrical polarization, and vice-versa [7]. The low number of materials displaying ME behaviour also did not support the proposed applications requiring specific physical properties and stability. In particular, the Curie temperature (T_c_) was below room temperature and the discrete compositions with limited possibilities for tuning the ME response were some of the Achilles heels in this area. Additionally, there were also limited techniques for the detailed/reproducible study of the ME behaviour [7].

The first ME composite material was produced in the 1970s by combining piezoelectric and ferroelectric BaTiO_3_ and piezomagnetic and ferromagnetic CoFe_2_O_4_ [8]. Despite the improvements on composites that allowed ME coupling to occur at room-temperature, the ME response was never above ≈ 100 mV·cm^−1^·Oe^−1^, due to inconsistencies in chemical reactions during the sintering process, low resistivity of the magnetostrictive phase, and induced eddy currents by the applied AC voltage. Also, dispersion problems in nanocomposites, mechanical defects limiting the mechanical coupling, and difficult on aligning the electric dipoles were issues still to be solved [7].

Only in the 1990s was interest on the ME effect renewed with the introduction of new ME interactions and degrees of freedom in designing ME compounds, innovative experimental techniques and optimized theoretical tools [9]. The experiments allowing one to tune new parameters, such as stoichiometry and the microstructure of the ME materials were carried out to produce large ME response, orders of magnitude higher than the one in single-phase materials at room temperature [7].

**Figure 2:**
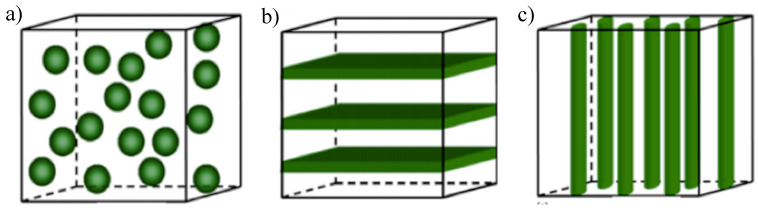
The schematic illustration of three bulk composites with different connectivity: (**a**) (0–3) particulate composite; (**b**) (2–2) laminate composite; (**c**) (1–3) fiber/rod composite. Reproduced with permission from [2].

The magnetic and piezoelectric phases in ME composites can be divided based on their connectivity types in (0–3) particulate composite; (2–2) laminate composite and (1–3) fiber/rod composite (Figure 2) [2].

In (0–3) particulate composites, a high concentration of magnetic particles needs to be dispersed into the selected piezoelectric matrix. The properties of the composite can be easily tailored by selecting the constituent phases, the particle sizes, and processing parameters [10].

In (2–2) laminate composites, the piezoelectric and magnetostrictive phases are often joined by a coupling agent, leading to an elimination of the leakage current, resulting on a superior ME performance [11]. Such laminates can be arranged in different shapes and geometries, including discs, squares, rectangles, and rings, with different dimensions. They can be arranged as unimorphs and bimorphs as well as bilayered and multilayered structures [12].

Regarding (1–3) fiber/rod composites, the magnetic phase can also lead to leakage currents during polarization [2], and very few attempts have been made to fabricate this type of bulk ME composite. To overcome this, (1–3) fiber/rod composites typically consist of three phases: PE bulk, magnetostrictive (MS) material and insulating polymers [13].

The first ME using a laminar topology was fabricated in 2001 by sandwiching a Lead zirconate titanate (PZT) disc between two Terfenol-D discs connected with silver epoxy, avoiding the previously reported problems in ME particulate composites, such as low ME response and leakage currents [7]. This laminate achieved a ME coupling coefficient of 4.68 V·cm^−1^·Oe^−1^ with a 4.2 kOe DC magnetic field at 1 kHz, exceeding the values obtained in ME particulate composites at the time [8].

These approaches and promising findings led to an increase research activity in the subsequent years, being a part of this research focused on substituting the ceramic piezoelectric materials of the ME particulate composites, by insulating piezoelectric polymers to overcome technical problems such as fragility and high dielectric losses, that lead to low output voltages [14].

**Figure 3:**
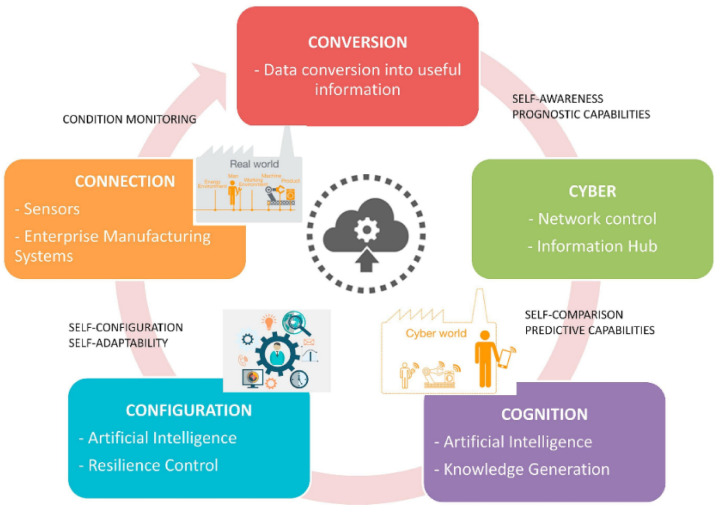
The model for Industry 4.0. Reproduced with permission from [15].

The polymer-based ME composites strategy offered a new approach for developing new applications with easy production at low temperatures and additive manufacturing capability (inkjet printing and screen printing, among others), tuned mechanical properties for flexible devices, large area applications, low-cost and biocompatible devices [14], suitable for the internet of things (IoT) and Industry 4.0. Such IoT devices demands on optimized performances, low power consumption and integrated applications can be achieved with ME materials (Figure 3).

The main experimental methods for measuring the ME coefficient are the static, quasi-static, dynamic and pulsed dynamic methods. For the dynamic method, the sample is subjected to the action of a superimposed AC field in a variable DC magnetic field, which generates at the ends of the material a voltage response (ME signal) which permits to obtain the ME voltage coefficient (α_ME_) value indirectly through Equation (1):

(1) $$\alpha_{ME}=\frac{\Delta V}{t\cdot H_{AC}}$$

where Δ*V* is the generated voltage, *t* the thickness of the piezoelectric material and *H_AC_* the intensity of the *AC* magnetic field.

This method had a great reception since it reduces the problems of charge accumulation at the edge of the sample, and for such reason in the most used in the literature [16].

ME systems converting magnetic energy into electrical output (or vice-versa) can increase the productivity and efficiency of resources [17]. Additionally, information and communication technologies allow ME smart products with embedded sensors, actuators, processing units, connected via internet enabling functionalities for monitoring, control, real time optimization and autonomy [18,19].

**Figure 4:**
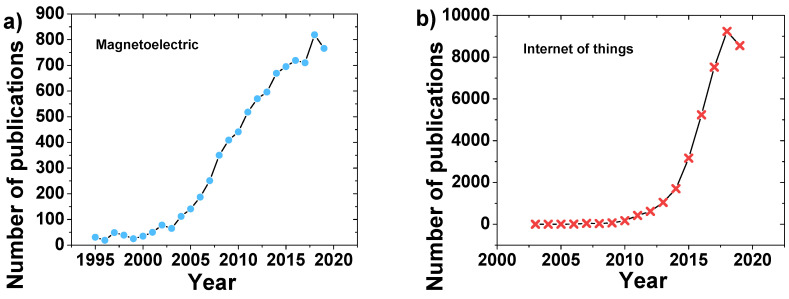
The publications per year according to the Web of Science database (7/2020) with the key words (**a**) magnetoelectric; and (**b**) internet of things [20].

Thus, the research on ME materials associated with IoT in the industry 4.0 context, taking advantage of the developments achieved over the past years and in both areas (Figure 4), can lead to new applications that enable monitoring and real time optimization and autonomy, by the introduction of self-sensing magnetic sensors and actuators for real time sensing, monitoring and response, enabling at the same time energy harvesting from the environment. Additionally, ME devices make possible to enhance real time communication with better tuned antennas.

This work aims to make a time travel through three centuries, starting in the pioneer studies regarding single-phase ME, until the implementation of ME materials within the 4.0 paradigm, passing by through a discussion concerning the main problems and future perspectives of the ME area.

## 2. Single-Phase ME

As previously shown, single-phase multiferroic materials are characterized by the intrinsic characteristic of presenting at least two ferroic orders [1]. The ME response is observed as an intrinsic effect typically at low temperatures (<10 K), that can be used for applications in data storage, spintronics or memories [2]. Shalini et al. [21] used a standard solid-state reaction method [22] by taking stoichiometric quantities of K_2_CO_3_, Na_2_CO_3_, Nb_2_O_5_, Fe_2_O_3_ MnO_2_, mixed in an agate mortar and calcined at 1373.15 K for two hours, creating the multiferroic ceramic K_0.5_Na_0.5_ [Nb_1−x_Fe_x/2_Mn_x/2_]O_3_ (x = 0.025, 0.05, 0.075, 0.10) [21]. This work verifies the behaviour in alkali niobate-based ferroelectric called potassium sodium niobite (KNN), a lead-free ceramic through the substitution of transition elements. This multiferroic ceramic material achieved a longitudinal ME coefficient (α_ME_) value of 3.45 mV·cm^−1^·Oe^−1^ with an H_DC_ of 1000 Oe and H_AC_ of 60 Oe [21].

**Figure 5:**
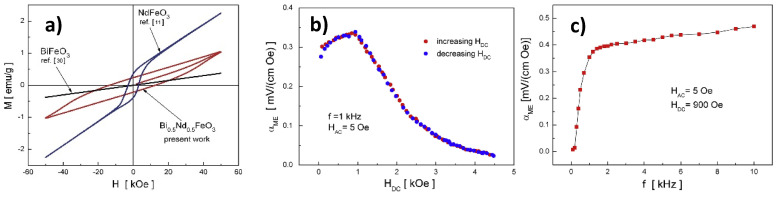
(**a**) Magnetic field dependence of the magnetization for the Bi_0.5_Nd_0.5_FeO_3_ solid solution. ME coupling coefficient variation with: (**b**) static magnetic field H_DC_ and (**c**) frequency f of sinusoidal magnetic field H_AC_ for Bi_0.5_Nd_0.5_FeO_3_ sample. Reproduced with permission from [23].

Pikula et al. [23] prepared a Bi_0.5_Nd_0.5_FeO_3_ solid solution using a standard solid-state reaction method. The stoichiometric mixture of oxide powders was grinded in a planetary ball mill for 24 h. The obtained mixture was consolidated into pellets and calcined at 1023 K for 10 h. Then, the material was crushed and milled in ethyl alcohol. After drying, the ceramic was again formed into pellets under a pressure of 60 MPa and then sintered at 1273 K during 24 h [23]. The samples were evaluated in a frequency interval from 100 Hz to 10 kHz with a applied H_DC_ of 0.9 kOe, achieving a maxim ME coefficient of 0.46 mV·cm^−1^·Oe^−1^ at 10 kHz (Figure 5) [23].

**Figure 6:**
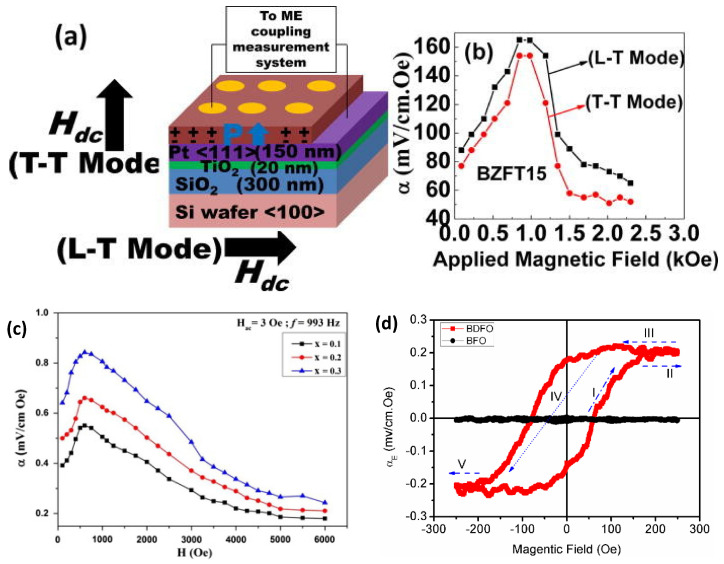
(**a**) The schematic representation of the experimental conditions; and (**b**) M–E coupling coefficient versus applied magnetic field measured in both in-plane magnetized-out of plane polarized configuration (L–T) and out of plane magnetized-out of plane polarized (T–T) modes. Reproduced with permission from [25]. (**c**) ME coefficient for a H_AC_ = 3 Oe at 993 Hz [26]. (**d**) MEP (H) hysteresis loops displayed at 300 K, showing the variations of the ME coefficient as a function of the applied magnetic field for of BDFO (red) and BFO (black). Reproduced with permission from [28].

Based on a similar procedure, Dabas et al. [24] reported a Mn-doped BiFeO_3_ ceramic. The material was prepared in the required stoichiometric proportion and ground in an agate pestle and mortar, mixed in an acetone medium. This mixture was ground until the acetone dried off, then transferred to a crucible for calcination at 973.15 K for 7 h. The mixture was pressed in a hydraulic press to obtain pellets of 10 mm of diameter by 1 mm of thickness. As a final step the pellets along with the powder mixture were sintered at 1093.15 K for 9 h, producing samples with 1, 3 and 5 molar percentage of Mn [24]. The ME coefficient of the samples with 1% Mn and 3% Mn is almost constant due to low amount of Mn, but in the 5% Mn one, a variation is observed for magnetic fields larger than 2000 Oe, leading to ME coefficient of 3.36584 mV·cm^−1^·Oe^−1^ at 2696 Oe, higher than the one for 1% Mn (≈ 0.170 mV·cm^−1^·Oe^−1^ at 2993 Oe) and 3% Mn (0.67 mV·cm^−1^·Oe^−1^ at 2792 Oe) samples [24]. Kumari et al. [25] developed a polycrystalline thin film by PLD of BaZr_0.05_(FexTi_1−3x/4_)_0.95_O_3_ grown on Pt/TiO_2_/SiO_2_/Si substrate (Figure 6a)). The ME coefficient at room temperature was ≈165 mV· cm^−1^·Oe^−1^ at 900 Oe for the x = 0.015 sample, Figure 6b) [25]. Joginder et al. [26] synthesized polycrystalline single phase Bi_4−x_Sm_x_Ti_3−x_Fe_x_O_12±δ_ (0 ≤ x ≤ 0.3) ceramics with a room temperature ME coefficient of 0.84 mV·cm^−1^·Oe^−1^ for x = 0.3 at 993 Hz with an applied AC field of 3 Oe, Figure 6c). The ME coupling appears through stress/strain mediated interaction between electric and magnetic sub-lattices [26].

Liu et al. [27] reported an effective ME coupling in cubic ferrimagnetic spinel LiFe_5_O_8_, showing a hysteretic ME signal at room temperature [27]. The LiFe_5_O_8_ powders, α-Fe_2_O_3_ and Li_2_CO_3_ were milled and heated at 1073.15 K for 5 h. The prepared powder was grounded and pressed into pellets with 6 weight percentage (wt.%) polyvinyl alcohol as binder, then sintered at 1223.15 K for 5 h, and coated with silver electrodes. This work demonstrated a maximum ME coefficient of 2 mV·cm^−1^·Oe^−1^ at a temperature of 120 K [27].

Ruth et al. [29] reported lead-free Na-deficient single-phase sodium bismuth titanate perovskite Na0.42Bi0.52Ti1.005O_3_ ferroelectrics. The material has shown a self-bias ME coefficient around 4.18 mV·cm^−1^·Oe^−1^ (H_AC_ = 1 Oe at 1 kHz) at zero DC magnetic field and room temperature [29].

Xue et al. [30] developed a solid solution of single-phase ME 00.6BiFeO_3_-0.1LaFeO_3_-0.3PbFeO_2.5_ based on a mixed oxide solution of BiFeO_3_, LaFeO_3_, and PbFeO_2.5_. These powders were mixed, ball milled, ground, calcinated and added to polyvinyl alcohol (PVA) 7 wt.% forming pellets. These pellets were sintered at 1 223.15 K for 2 h then coated with silver electrodes and annealed at 823.15 K for 30 minutes [30]. The single-phase materials presented a ME coupling of approximately 120 mV·cm^−1^·Oe^−1^ [30].

Lakshmi et al. [31] synthesized multiferroic ceramic BiFeO_3_ co-doped with aliovalent Nb, Mn and Mo at the Fe site by sol-gel [31,32,33]. Bi(NO_3_)_3_·5H_2_O, Fe(NO_3_)_3_·9H_2_O, Er(NO_3_)_3_·H_2_O, Nb_2_O_5_, Mn(NO_3_)_2_·H_2_O, MoO_6_ were mixed in distilled water and stirred at 400 rpm until homogenization. Then, the mixture was heated at 353.15 K on a hot plate until a gel was formed. The gel was converted into powders by auto-combustion, which was then ground and annealed at 873.15 K for 12 h. The pellets of 8 mm were prepared from the annealed powders by a hydraulic press under 6 tons pressure and sintered at 1073.15 K for 12 h [31]. The results of BiFeO_3_ doped with Er and Nb showed a maximum ME coupling coefficient of 0.22 mV·cm^−1^·Oe^−1^ at 13 mT [31].

Luo et al. [20] fabricated Bi_4_SmFeTi_3_O_15_ thin films coated on (111)Pt/Ti/SiO_2_/Si substrates by the sol–gel method. The precursor solution was constituted by high-purity bismuth nitrate (Bi(NO_3_)_3_·5H_2_O 98%), samarium oxide (Sm_2_O_3_ 99.9%), iron acetylacetonate (C_15_H_21_FeO_6_ 98%) and titanium *n*-butoxide Ti(C_4_H_9_O_4_) (99%). The ME coefficient observed at room temperature was of 41.16 mV·cm^−1^·Oe^−1^ at 0.9 T [20]. Zhao et al. [34] developed a room temperature bismuth-layer-structured ferroelectric Bi_5_Ti_3_FeO_15_ thin film grown by pulsed laser deposition (PLD) technique [35] on Pt/Ti/SiO_2_/Si substrates. The achieved ME coupling at room temperature was of approximately 400 mV·cm^−1^·Oe^−1^ at a zero bias magnetic field (H_AC_ = 5 Oe) [34]. Pan et al. [28] produced a room temperature ME multiferroic based on BiFeO_3_, Bi_0.88_Dy_0.12_Fe_0.97_Ti_0.03_O_3+δ_, prepared via a co-precipitation process with the starting constituents Bi(NO_3_)_3_·5H_2_O, Dy(NO_3_)_3·_5H_2_O and Fe(NO_3_)_3_· 9H_2_O. An electric field of 80 kV·cm^−1^ was applied at room temperature to pole the sample and the ME effect at room temperature reached approximately 0.23 mV·cm^−1^·Oe^−1^ at 250 Oe, Figure 6d). The response at low fields could potentially bring BiFeO_3_ materials closer to practical applications in electronics and spintronics devices were the dipoles can be tuned by a low magnetic field [28].

**Figure 7:**
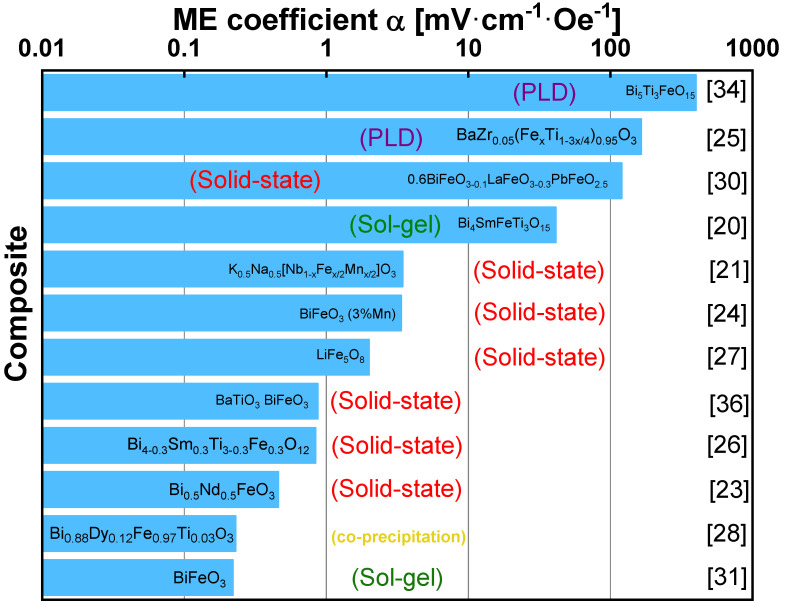
Some representative single-phase materials and their corresponding ME voltage coefficient and fabrication process.

Yang et al. [36] developed single-phase multiferroic ceramics of (1 − x) BaTiO_3_ − x BiFeO_3_ (BT − x BFO) synthesized by solid-solution method that exhibited an ME coefficient of 0.87 mV·cm^−1^·Oe^−1^, providing a possibility of developing electrically or magnetically tunable thin-film devices. The reported single-phase ME materials, as well as their production technique are summarized in Figure 7, being observed that the ME voltage coefficient can assume a broad range of values, from less than 1 mV·cm^−1^·Oe^−1^ up to almost 1 V·cm^−1·^Oe^−1^.

## 3. Ceramic-Based ME

Ceramic ME composites consist of a ferroelectric oxides and magnetic oxides (mainly ferrites) combination, and they reveal ME coefficients three orders of magnitude higher than ceramic-based ME materials [37]. Improved piezoelectric and ferroelectric properties in ceramics can be achieved through:

- selection of the composition (preferably near morphotropic phase boundary (MPB) or polymorphic phase transition (PPT)) and modification by doping;
- microstructure design via domain engineering and texturing [38].

Lead zirconate titanate (PZT) [39] has been very often used as the ferroelectric phase in ME composites due to its remarkable piezoelectric (PE) effect. At the same time, ferrites are used as magnetostrictive components due to their high magnetostrictive (MS) performance [11], being found reports employing cobalt ferrite (CoFe_2_O_4_) [40], nickel ferrite (NiFe_2_O_4_) [41] and Ni 0.8Zn 0.2Fe_2_O_4_ as magnetic phase [42]. Lopatina et al. [43] prepared and investigated PZT/ferrite composites of different connectivity. The highest value of the ME coefficient (110 mV·cm^−1^·Oe^−1^) with the bias magnetic field H*_o_* = 0.9 kOe) were found in sintered mixtures of PZT powders with ferrite and sliced materials with 35–55 wt.% of ferrite.

The main disadvantage of PZT is the presence of lead, which is being replaced by lead-free dielectrics. It is also reported that PZT films exhibit high acoustic loss at high frequency range [44]. For these reasons, studies have been carried out to find lead free materials comparable in terms of properties, such as BaTiO_3_, Na_0.5_Bi_0.5_TiO_3_, K_0.5_Bi_0.5_TiO_3_ or Na_0.5_K_0.5_NbO_3_ [45,46,47].

It should be noted that ferrites may lead to leakage currents that, consequently, can cause deterioration of the composite insulation. This type of material is processed at high temperatures which can lead to a lower ME coefficient than the one that is theoretically predicted, due the inherent preparation problems, such as thermal expansion and atomic interfacial interdiffusion reactions [48,49]. Chemical solution processing and novel sintering techniques such as spark-plasma sintering (SPS) and microwave sintering have been employed to produce the particulate ceramic composites [50,51,52]. Other magnetostrictive materials, such as Tb_(1-x)_Dy_x_Fe_2_ (Terfenol-D) or Metglas are of great interest for practical applications [53].

Duong et al. [54] reported that (2–2) laminate composites with 50 wt.% CoFe_2_O_4_ and 50 wt.% of BaTiO_3_ revealed an enhanced magnetostrictive and piezoelectric coupling compared to the same materials in (0–3) particulate composites, with a ME coefficient of 5.5 and 4.2 mV·cm^−1^·Oe^−1^ for longitudinal and transverse measurements, at the field 2300 and 940 Oe, respectively. Hang et al. [55], also showed that (2–2) laminate composites of BTO/CFO exhibit higher ME coefficients than the (0–3) particulate composite (135 mV·cm^−1^·Oe^−1^and 35 mV·cm^−1^·Oe^−1^, respectively, at a DC magnetic field of 2600 Oe and a frequency of 1 kHz), related to the reduction of dielectric losses. Another strategy to reduce ME signal loss is the placement of internal electrodes (e.g., Ag, Ni, and Ag-Pd) between the PE and MS layers [48,56].

**Figure 8:**
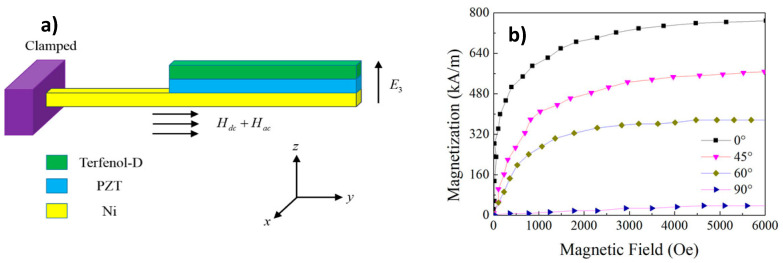
(**a**) The configuration of laminated Terfenol-D/PZT/Ni composite cantilever; (**b**) magnetization of Terfenol-D with bias field at different orientation angles. Reproduced with permission from [56].

Among the three main connectivities of ME composites, (2–2) laminate structures present the highest ME coefficient due to the use of MS alloys since 2001 [13]. Also, on ceramic-based composites, Terfenol D has been used due to its ability to combine low frequency operation and high energy density with high magnetostriction (1000 ppm at fields of 5 kOe) [57]. Both theoretical and experimental results have been reported [58,59] (Figure 8). However, Terfenol-D-based ME composites present low permeability and high saturation field (700 kA/m), and because of that, it is not appropriate for low magnetic field applications. Recent research on ME composites has been focused on the search for new magnetostrictive materials having balanced soft magnetic and magnetostrictive properties. FeBSiC alloys (Metglass) in the form of amorphous ribbons developed by a fast solidification process, allow for fast magnetization and demagnetization, showing high permeability and low coercivity and saturation field [60].

**Figure 9:**
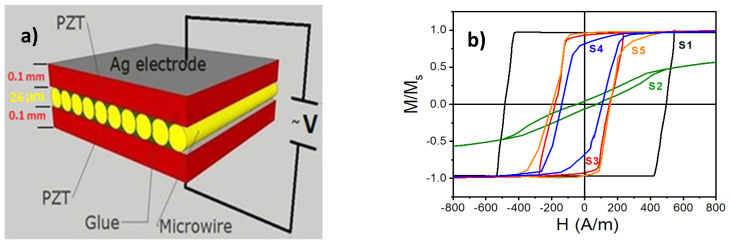
(**a**) The sketch of the structure of ME composites with microwires; (**b**) Hysteresis loops of individual microwires with composition of Fe_77.5_B_15_Si_7.5_ in as-cast state (S1) and after different treatments (S2–S5: As-cast and then annealed (S2); As-cast and glass-coat removed (S3); As-cast, annealed, and then glass-coat removed (S4); As-cast, glass-coat removed, and then annealed) [61].

Amirov et al. [61] proposed a new approach for a ME sandwich structure comprising glass-coated amorphous Fe_77.5_B_15_Si_7.5_ microwires as magnetostrictive layer and PZT as piezoelectric phase with a reported ME coefficient of 100 mV·cm^−1^·Oe^−1^ (Figure 9). This maximum ME response was observed in weak magnetic fields about 3 Oe at electromechanical resonance conditions (50–60 Hz). The authors stated that this approach can be used for the design of self-biased ME composites that provide large ME coupling under an external AC magnetic field in the absence of a DC magnetic field. Greve, et al. [62] fabricated thin film ME (2–2) composites consisting of aluminum nitride (AlN) and amorphous (Fe_90_Co_10_)_78_Si_12_B_10_ layers by magnetron sputtering on Si (100) substrates.

Upon magnetic field annealing an exceptionally high ME coefficient of 737 V·cm^−1·^Oe^−1^ at the mechanical resonance of 753 Hz and 3.1 V·cm^−1·^Oe^−1^ out of resonance at 100 Hz, at 6 Oe was demonstrated. These are the highest ME coefficients in thin film composites reported ever [62].

(x)BaTiO_3_–(1−x)Co_0.7_Fe_2.3_O_4_ ME nanocomposite ceramics with x = 0.0, 0.25, 0.50, 0.75, 1.00 were successfully designed and fabricated by sol-gel route by Gaikwad et al. [63]. The resulting ME voltage coefficient, 7.7 mV·cm^−1^·Oe^−1^, makes the material suitable for storage device applications.

By using an unsymmetrical bi-layered Metglas/Pb(Zr,Ti)O_3_ ME composites with multi-push pull configuration, Gao et al. [64] obtained a ME voltage coefficient of 250 V·cm^−1^·Oe^−1^, on which the resonance frequency can be varied from 70 Hz to 220 Hz, allowing the authors to develop a magnetic field energy harvesters capable of harvesting energy generated by electronic instruments working on a 60 Hz AC power supply. Using the same materials, Palneedi et al. [65] deposited PZT on a Metglas foil by a granule spray in vacuum process at room temperature, followed by its localized annealing with radiation from a continuous-wave 560 nm ytterbium fiber laser. As a result, a colossal off-resonance ME voltage coefficient (3 V·cm^−1·^Oe^−1^—two orders of magnitude larger than previously reported) output from the PZT/Metglas film-composites was achieved.

Keeping the focus on PZT-based materials and changing the magnetostrictive phase to Terfenol-D, Wen et al. [66] reported a ME coefficient of 10 V·cm^−^1·Oe^−1^ suitable for broadband magnetic field sensors. A lead-free ME laminate composite consisting of thickness-polarized piezoelectric Mn-doped Na_0.5_Bi_0.5_TiO_3_-BaTiO_3_ single crystal and length-magnetized magnetostrictive Tb_0.3_Dy_0.7_Fe_1.92_ alloy (L-T mode) have been fabricated by Wang et al. [67], exhibiting a linear ME response of 1.32 V·cm^−1·^Oe^−1^ that opened up the possibility for environment-friendly magnetic sensors.

Amorín et al. [68] reported an in-depth study of the local material properties across the interfaces of 0.36BiScO_3_-0.64PbTiO_3_/NiFe_2_O_4_ multilayer ceramic composites, processed by spark plasma sintering of nanocrystalline powders being obtained a ME voltage coefficient of 100 mV·cm^−1^·Oe^−1^.

**Figure 10:**
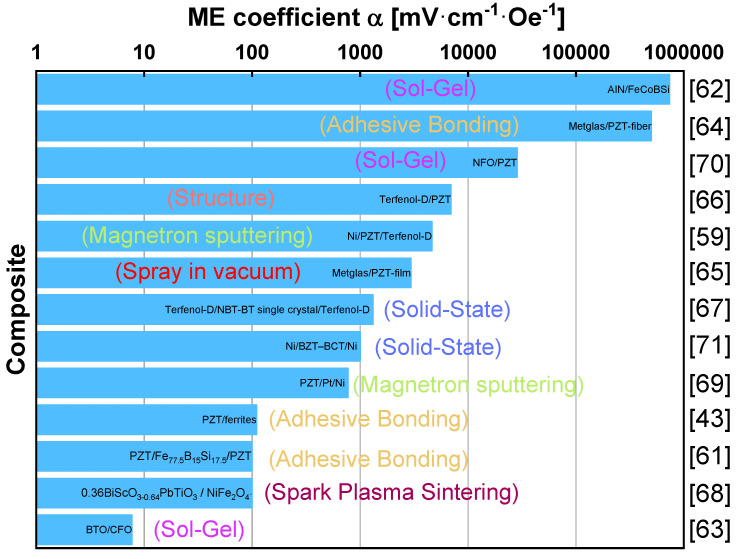
Some representative ceramic-based ME materials together with the ME coefficients, processing techniques, and main application areas.

Polycrystalline Pt thin films of different thicknesses (0–75 nm) were introduced using magnetron sputtering in PZT (400 nm in thickness))/Pt/Ni multiferroic film heterostructures by Feng et al. [69], aiming an optimized transfer efficiency of magnetostrictive strain from the bottom Ni foil to the top PZT film. It was obtained a direct ME voltage coefficient of 772 mV·cm^−1^·Oe^−1^. ME composite ceramics of NiFe_2_O_4_ and PZT were also synthesized by a simple in situ processing based on a sol-gel method followed by a conventional sintering, exibiting a ME voltage coefficient of 28.5 V·cm^−1·^Oe^−1^ [70]. A strong and anisotropic ME effect (1 V·cm^−1·^Oe^−1^) was obtained in composites of magnetostrictive Ni and solid-state grown lead-free piezoelectric 82BaTiO_3_ −10BaZrO_3_ −8CaTiO_3_ single crystals [71] enabling the realization of customized ME effects in composites. The reported ceramic-based ME materials, as well as their production technique are summarized on Figure 10, being observed that the ME voltage coefficient can assume a broad range of values, from less than 10 mV·cm^−^^1^·Oe^−^^1^ until almost 1 kV·cm^−^^1·^Oe^−^^1^.

## 4. Polymer-Based Magnetoelectrics

Despite the polymer-based ME materials presenting a lower ME coefficient, they are increasingly being used as they allow overcoming some of the above-mentioned problems associated to the ceramic-based ME materials such as fragility, rigidity and high-cost and also due to their facile, scalable and low-temperature manufacturing methods. The capability to be fabricated in a diversity of forms and shapes, their printability and, in some situations, their biocompatibility are also taken to important advantages [10,72]. Among the different methods that are reported for polymer-based ME nanocomposite films development, solution casting is among the most used one [73].

Andrade et al. [74] demonstrated that the introduction of Gd_5_(Si_2.4_Ge)_1.6_ (GSG) into an electroactive matrix (PVDF) gives rise to a ME coupling and a multicaloric effect. A large ME response of 2.2 V·cm^−1·^Oe^−1^ (H_DC_ = 5 kOe at 305 K) was observed for 12 wt.% of filler concentration. It was concluded that the ME effect in PVDF/GSG composites is a result of the elastic cooperation between magnetostrictive and piezoelectric components behavior with a contribution from a thermal mediation arising from the components magnetocaloric and pyroelectric features.

Polyurethane (PU) has been also used as polymeric matrix. Guyomar et al. [75] prepared particulate polymer composites by solution casting, consisting of PU/Terfenol-D, PU/Fe_3_O_4_ and PU/Nickel. It was concluded that whatever the filler type (Terfenol-D, Fe_3_O_4_ or Nickel), the micro composites show a ME response and that the magnetostrictive property of the material does not have a direct influence on the ME effect since ME sensitivity is DC field independent and the ME polarization coefficient *α*_p_ show close values in ac fields for all types of polymer fillers (Table 1).

**Figure 11:**
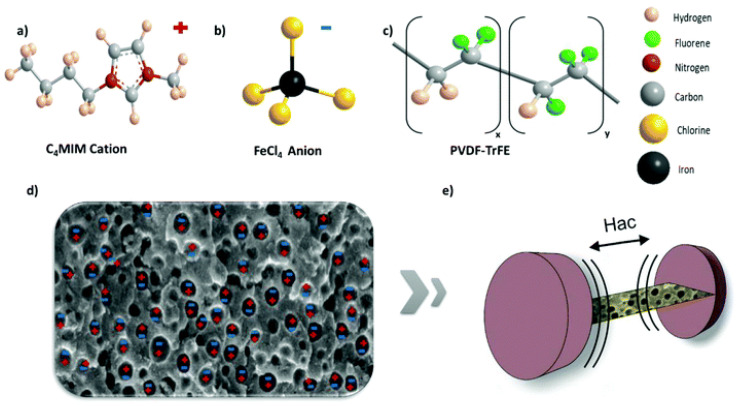
The MI nanocomposite and experimental set-up for the measurement of the MI effect. (**a**) Schematic representation of the [C_4_mim] + cation. (**b**) Schematic representation of the [FeCl_4_]—anion. (**c**) Illustration of the P(VDF-TrFE) monomer structure. (**d**) Profile chart of the MI composite. (**e**) Schematic view of the system for MI voltage measurement. Reproduced with permission from [77].

Recently, polymer-based ionic liquid composites, in the form of films and fibers have been processed, with focus on the influence of the cations or anions on the physical–chemical properties of the composites and in the improvement of electromechanical actuators [76]. Correia et al. [77] demonstrated the concept of exploring magnetic ionic liquids (MIL), such as [C_4_mim][FeCl_4_], in polymer-based ME nanocomposites, suitable for low-field magnetic sensing devices (Figure 11).

The novel MI nanocomposites produced using P(VDF-TrFE) as the passive porous material, showed a giant α_ME_ coefficients of 10 V·cm^−1·^Oe^−1^, at a H_AC_ intensity of 2 Oe and H_AC_ frequency of 10 kHz. This response includes a magnetically triggered ionic/charge movement within the porous structure of the polymer, being this a novel phenomenon never experimentally observed or explored in ME composites before.

Noticeably, enhanced values of α_ME_ have been found in laminated composites consisting of magnetostrictive and piezoelectric layers glued together, since the beginning of the millennium [78]. This type ME composites show ME voltage coefficients of up to 1000 × larger than any other type of ME materials, leading to the designation of giant ME effect. The elastic coupling in ferromagnetic/ferroelectric polymer-based laminates was first reported by Mori in 2002 [79]. From the different polymers used for the development of ME laminates, polyvinylidene fluoride (PVDF) and its copolymers are the most popular ones; nevertheless, it is possible also to find several works with diamines [80].

**Figure 12:**
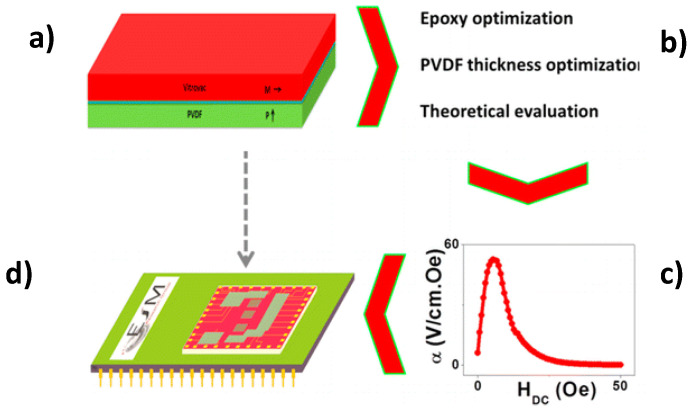
The schematic representation of the Vitrovac/Epoxy/PVDF composite (**a**); optimization process (**b**) and ME response (**c**) pave the way for its incorporation into technological applications such as magnetic sensors (**d**). Reproduced with permission from [82].

Concerning to the magnetostrictive component of laminates, there are three materials responsible for almost 90% of the work carried out on polymer-based ME laminates: Metglas, VITROVAC, and Terfenol-D [81]. Tri and bi-layered ME flexible composite structures of variable geometries and sizes with magnetostrictive VITROVAC and piezoelectric PVDF layers were fabricated through direct bonding. Silva et al. [82] reported the effect of the bonding layer type and piezoelectric layer thickness on the ME response of layered (PVDF)/epoxy/Vitrovac composites (Figure 12).

An increase of the ME voltage coefficient from 45 V·cm^−1·^Oe^−1^ to 53 V·cm^−1·^Oe^−1^ was verified with increasing PVDF thickness from 28 µm to 110 µm and a reduction of the ME voltage coefficient from 53 V·cm^−1·^Oe^−1^ to 6 V·cm^−1·^Oe^−1^ with increasing epoxy Young modulus from 2.7 × 10^8^ Pa to 9.0 × 10^9^ Pa [82]. Jing et al. [83], prepared, by a hot-pressing method, a polymer-matrix composite of (CFO/CNT/PVDF)/P(VDF-TrFE)/(CFO/CNT/PVDF) with a P(VDF-TrFE) layer sandwiched between two layers of CFO/CNT/PVDF mixtures. The α_ME_ of the polymer-matrix composites increases with increasing volume fraction of CFO particles. The self-biased and peak value α_ME_ of the composites reach up to 16.7 mV·cm^−1^·Oe^−1^ and 25.8 mV·cm^−1^·Oe^−1^, respectively, at approximately ± 1000 Oe, at low frequency of 1 kHz.

**Figure 13:**
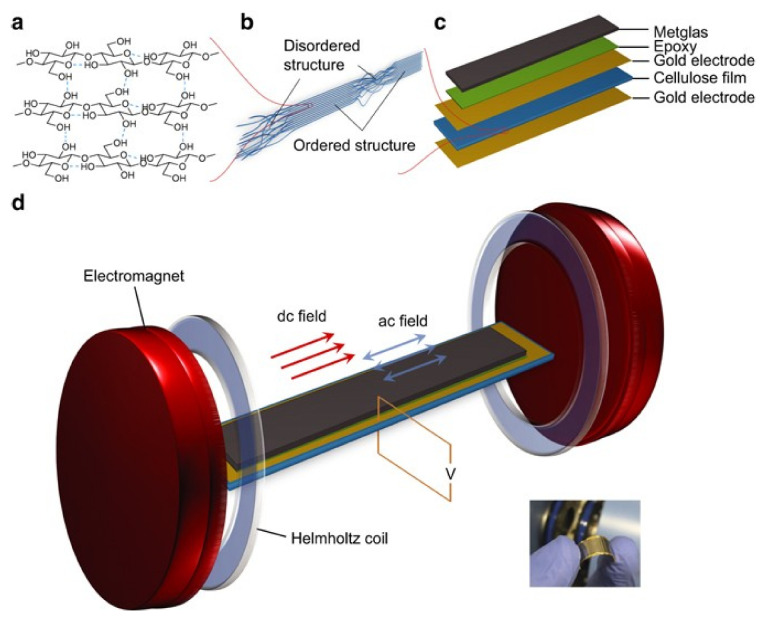
(**a**) The scheme of cellulose crystal II; (**b**) Illustration of cellulose fibril alignment at the cross-section of cellulose film; (**c**) Schematic of cellulose-based ME laminate structure; (**d**) Schematic view of the bulk system for ME voltage measurement. Reproduced with permission from [84].

PVDF is considered as the “material of merit” for these applications, however, Zong et al. [84], stated that there is an enormous potential in other polymers to bring significant added-value properties and function to ME composite devices. For this reason, it was reported the development of ME composites based on the natural biopolymer, cellulose. To fabricate cellulose-based ME composites, the authors prepared laminate (bilayer) structures comprising Metglas and cellulose films (Figure 13), enabling two-phase strain coupling for enhanced ME response.

The cellulose-based ME laminate composite produced a substantial ME coefficient of approximately 1.41 V·cm^−1·^Oe^−1^, at H_DC_ = 4.2 Oe [84]. Although laminated ME materials exhibit a higher (three orders of magnitude) ME response, nanocomposites offer other benefits such as cost effectiveness, high control of the process parameters and scalable production with good reproducibility [85].

Innovatively multifunctional ME composites of poly(vinylidene fluoride-co-trifluoroethylene) P(VDF-TrFE)/CoFe_2_O_4_ were spray-printed [86], being observed a ME voltage coefficient of 21.2 mV·cm^−1^·Oe^−1^, demonstrating the suitability of these materials for cost-effective and large-scale sensor/actuator applications, namely in aerospace, automotive and recreational products.

ME material were also printed by the screen-printing technique [87] by using P(VDF-TrFE as the piezoelectric phase and poly(vinylidene fluoride)(PVDF- CoFe_2_O_4_) as the magnetostrictive phase. The reported ME coefficient was 164 mV·cm^−1^·Oe^−1^ suitable for printed electronics, sensors, actuators, and energy harvesters. The work of Martins et al. [88] also focused on the development of a new type of ME polymer nanocomposites that exhibited a tailored ME response at room temperature. The authors developed multiferroic nanocomposites based on three different ferrite nanoparticles, Zn_0.2_Mn_0.8_Fe_2_O_4_ (ZMFO), CoFe_2_O_4_ (CFO) and Fe_3_O_4_ (FO), dispersed in P(VDF-TrFE) matrix. ME results of the nanocomposite films with 10wt.% ferrite content revealed that the ME induced voltage increases with increasing DC magnetic field until a maximum of 6.5 mV∙cm^−1^∙Oe^−1^, at an optimum magnetic field of 0.26T, and 0.8 mV∙cm^−1^∙Oe^−1^, at an optimum magnetic field of 0.15 T, for the P(VDF-TrFE)/CFO and P(VDF-TrFE)/FO composites, respectively.

By producing P(VDF-TrFE)/BaTiO_3_ composites through a solvent casting method, Mayeen et al. [89] reported a ME voltage coefficient of 18.2 mV∙cm^−1^∙Oe^−1^ suitable for energy storage, harvesting, energy conversion. By depositing a PVDF solution of a Metglas substrate a ME voltage coefficient of 686 mV·cm^−1^·Oe^−1^ was obtained, that was used on a magnetic sensor with a sensitivity of 503.3 V·T^−1^ and correlation coefficient of 0.9994 [90].

Polymer-based ME materials were also tested for magnetic sensing by Reis et al. [91] who bonded a PVDF layer to a Metglas foil. The observed ME voltage response (30 V·cm^−1·^Oe^−1^) allied to a charge amplifier, an AC-RMS converter and a microcontroller with an on-chip analog-to-digital converter allowed 1.5 V·Oe^−1^ (15 kV·T^−1^) sensitivity and a 70 nT resolution, very attractive for applications such as Earth magnetic field sensing, digital compasses, navigation, and magnetic field anomaly detectors, among others. The same author also fabricated and characterized a high-performance polymer-based ME DC magnetic field AC/DC sensing device composed of PVDF and Fe_61.6_Co_16.4_Si_10.8_B_11.2_. The sensing device exhibited a ME response (250 V∙cm^−1^∙Oe^−1^), accuracy (99% for both AC and DC sensors), linearity (92% for the DC sensor and 99% for the AC sensor) and reproducibility (99% for both sensors) indicate the suitability of the sensor for applications [92].

Using other copolymer of PVDF-poly(vinylidene fluoride-hexafluoropropylene) [P(VDF-HFP)], Lu et al. [93] glued the polymer to a Metglas ribbon (2605SA1), being reported a 12 V cm^−1^∙Oe^−1^ ME voltage coefficient that followed linear relationship with the in situ poling electric field.

Using a dual approach (by experiments and modeling) Belouadah et al. [94], studied the phase switching phenomenon in ME laminate polymer composites, reporting the ME effect (≈900 MV∙cm^−1^∙Oe^−1^) observed in bi- and trilayered polymers consisting of PVDF and polyurethane (PU) filled with magnetically hard magnetite Fe_3_O_4_ or Terfenol-D magnetostrictive material. A good agreement between the simulated results and experimental data was obtained and it was found that phase switching characteristics are mainly influenced by the ME losses induced by magnetostriction losses. In particular, nanocomposites allow advanced production techniques, such as additive manufacturing, facilitating technology transfer to the industry and integration into devices [77]. Consequently, laminated composites present higher ME responses at lower magnetic fields whereas nanocomposites are characterized by improved production features. Therefore, the future of ME materials is closely related to the optimization of the ME response on nanocomposites or with the introduction of new effects to meet higher ME performances for a wide range of applications [73].

**Figure 14:**
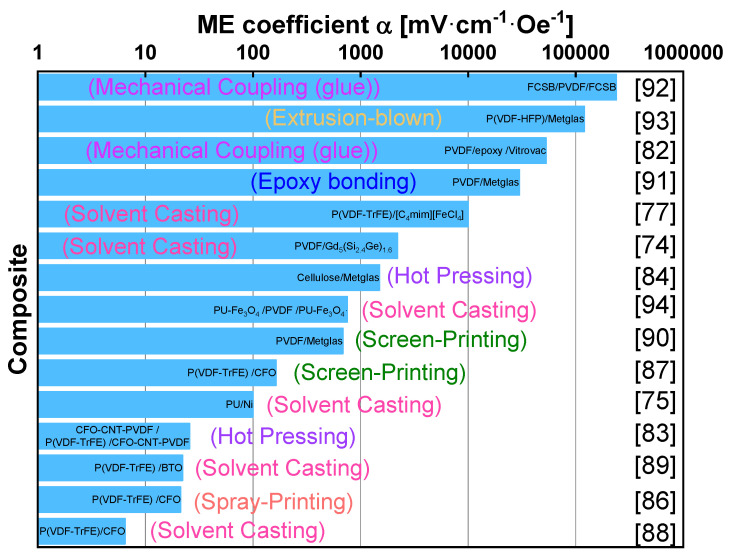
Some representative polymer-based ME materials, and their respective ME voltage coefficient.

The reported polymer-based ME materials, as well as their production techniques are summarized on Figure 14, being observed that the ME voltage coefficient can assume a broad range of values, from less than 10 mV·cm^−1^·Oe^−1^ up to almost 250 V·cm^−1^·Oe^−1^.

## 5. Applications in the 4.0 Context

In IoT, devices are wireless connected, through communications protocols, to the internet. Each device has a unique identifier that allows them to communicate with each other by sending or receiving data. The main concepts of an IoT system are the dynamic nature, the self-adapting ability, the self-configuration and the self-powered capacity [95]. ME devices have the capability to sense and harvest the ambient magnetic energy based on direct magnetic coupling. On the other hand, filters, tunable resonators and memories can capitalize the converse ME coupling, where the electric field controls the permeability, the spintronics or the magnetization [53]. Additionally, when compared to the piezoelectric materials, ME materials can harvest/sense magnetic energy and are more suitable for constitution of technical devices for wireless communication. Einstein has stated that any energy is coupled with gravitation. As a result, the ME materials can also support the acoustic wave propagation coupled with some gravitational phenomena. The original theory was developed in 2016 [96] and the possible hot application is the development of instant interplanetary communication [97].

Regarding energy generators Ghosh et al. [98], developed a flexible rollable magneto-mechano-electric nanogenerator (MMENG) for wireless IoT to capture parasitic magnetic noise native from electrical power transmission systems. The device was produced by combining magnetostrictive NiFe_2_O_4_ nanoparticles, with approximately 9 nm of diameter and P(VDF-TrFE) polymer and a ME coefficient of 11.43 mV·cm^−^^1^·Oe^−^^1^ was achieved. The device placed on a magnetic field of 1.7 × 10^−^^3^ T at 50 Hz from the power cord of a kettle, produce a peak-to-peak voltage (V_PP_) of 1.4 V and an output power density of 0.05 µW·cm^−^^3^. The signal was transmitted to a smartphone for demonstration of position monitoring for the integration of self-power sensors on implantable biomedical devices and human health monitoring sensory systems [98].

Yang et al. [99], reported a vibration energy harvesting multi-cantilever beam. The system is composed by a ME transducer by sandwiching one layer of PMNT between two layers of Terfenol-D. For the experimental setup, the authors used a vibration shaker connected to a function generator and an accelerometer mounted on the shaker. The output voltage was measured by a digital oscilloscope. The shaker acceleration was set to 0.2 g and the frequency swiped from 15 to 40 Hz. This system was capable of producing a power density of 0.2–0.56 mW·cm^−^^3^ under an acceleration of 0.2 g [99]. A PZT/ Ni unimorph cantilever with a NdFeB magnet as tip mass was developed by Lu et al. [100] for energy harvesting in wireless sensing applications. This harvester has a maximum power density of 270 µW·cm^−^^3^ at the resonance frequency of 50 Hz. The demonstration showed that the device was capable of harvesting energy from the ambient magnetic energy and powering commercial wireless temperature/humidity sensors [100].

**Figure 15:**
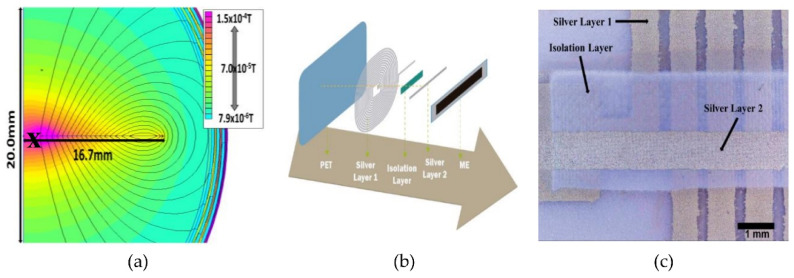
(**a**) The theoretical simulation of the AC magnetic field (in T) generated for a printed coil with a width of 750 and 250 μm spacing, 15 turns and a current (I) = 0.02 A. (**b**) Schematic representation of the printing process of the coils. (**c**) Coil printing detail obtained with a digital microscope. Reproduced with permission from [101].

The work on [101] reports on a magnetic proximity sensor combining printed technologies and a polymer-based ME laminate. This ME laminate was produced by direct bonding a commercial polarized PVDF film, with deposited contacts, and magnetostrictive Metglas layers magnetized along the length direction. The sensor was completed by printing a coil by screen printing technique with silver ink, Figure 15. The proposed device creates an AC magnetic field from the coil that is directed to the ME laminate and when a DC magnetic field is present, the sensor reacts with an increase or decrease of the peak-to-peak voltage proportional to the applied DC magnetic field. This sensor presents a resonance frequency of 13.2 kHz, and a ME coefficient of 50.2 V·cm^−^^1·^Oe^−^^1^ [101].

Friedrich et al. [102], developed a magnetic particle mapping (MPM), based on a ME sensor composed by Fe_70.2_Co_7.8_Si_12_B_10_ (FeCoSiB) and aluminium nitride fabricated MEMS for the detection for localizing magnetic particles in a medical context. The magnetic particles were biocompatible and were used for diagnostic/treatment of cancer and labelling and tracking cells. The proposed technique detected the nonlinear magnetic response at a magnetic excitation filed on a ME sensor in a freestanding cantilever topology with a low limit detection of 100 pT/(Hz)^0.5^ at 10 Hz using modulation techniques. The sensor exhibited a bandpass behaviour with a 10 Hz bandwidth and a resonance frequency of 7.55 kHz. With the use of a charge amplifier, the sensor presented a maximum sensitivity 18 kV/T. The MPM can be used as an alternative of more expensive techniques such as magnetic resonance imaging (MRI) or magnetorelaxometry imaging (MRX) [102].

In 2019 Ou et al. [103], developed a SrFe_12_O_19_/FeCuNbSiB/PZT self-biased ME cantilever sensor. The sensor has shown a large output voltage and sensitivity (198.91 mV·A^−1^ at 50 Hz) without DC bias magnetic field and is capable of detection steps of 0.01 A. This sensor can be used on the monitoring of power line consumption [103].

**Figure 16:**
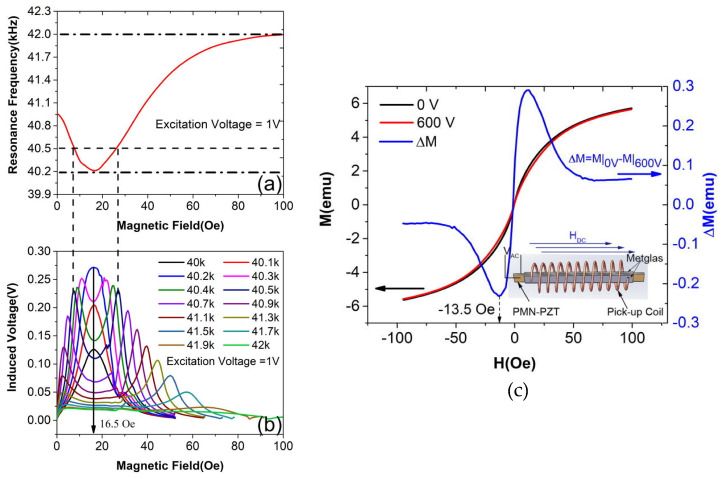
(**a**) The resonance frequency of the ME laminate as a function of applied H_DC_, (**b**) The induced voltage as a function of applied HDC and excitation frequency, (**c**) The M-H loop of the ME laminate with voltages of 0 V and 600 V applied to the piezoelectric crystal. ΔM as a function of external HDC is also given on the right. The inset shows the schematic of the ME heterostructure. Reproduced with permission from [104].

Chu et al. [104], reported a ME magnetic field sensor based in the control of the magnetization via an applied electric field with a ME laminate composed by Pb(Mg_1/3_-Nb_2/3_)O_3_-Pb(Zr, Ti)O_3_ (PMN-PZT) crystal, poled along the thickness direction and a pickup coil wound around the laminate. This laminate was electrically driven by the contacts deposited on the piezoelectric layer at the frequency of its first-order longitudinal vibration mode, and the induced voltage was captured by the pickup coil based on the law of electromagnetic induction, Figure 16. The ME laminate show a sensitivity of 3400 V/T, the induced voltage was mainly proportional to the excitation voltage. The sensor also exhibited a limit of detection of ≈115 pT at 10 Hz and 300 pT at 1 Hz, with a power consumption of 0.56 mW [104].

Xu et al. [105], developed a low frequency transmitter based on the capabilities of the ME resonance sensor. The ME laminate was composed by a piezoelectric layer (PZT-5A) at the core and a magnetostrictive layer on top and bottom (Metglas). This device exhibited higher efficiency compared to current loop antenna of the same size. The proposed device has successfully demonstrated the generation of an AC magnetic field by driving the piezoelectric phase at the resonance frequency (30 kHz). The device also revealed the capability to act as a receiver antenna [105]. Keeping focus on the antennas, NanoNeuroRFID is an ultra-compact implantable device composed by a ME antenna array that can harvest electromagnetic energy to power the device, sense quasi-static neuronal magnetic fields as small as 200 pT without direct contact to the tissue, communicate with an external transceiver and works from 10 to 100 MHz, where tissue loss is small [106]. The ME antenna based on FeGaB/AlN thin film exhibited a sensitivity of 2.475 Hz/nT and an ultra-low magnetic noise of 2.36 pT/Hz^1/2^ at 10 Hz. The device is composed by an array of ME antennas for increasing the output voltage [106].

**Figure 17:**
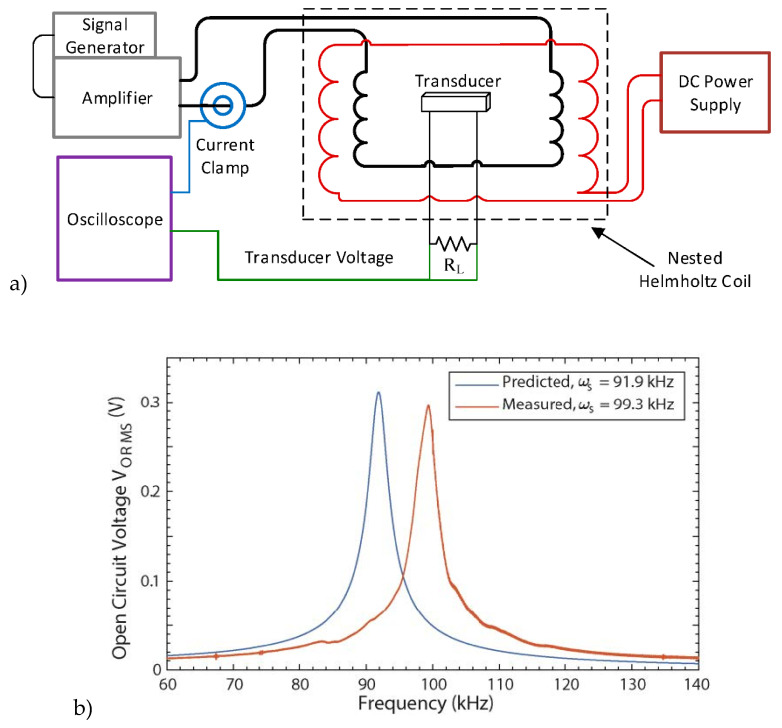
(**a**) The ME transducer experimental test setup diagram. (**b**) Metglas-PVDF open circuit voltage vs. frequency. Reproduced with permission from [107].

Rupp et al. [107], developed a wireless power receiver (Figure 17a)) for wearables and implantable applications based on a ME laminate composed of PVDF/Metglas with a very small footprint, smaller than 2 mm^3^. The presented device was able to produce a higher power density when compared to conventional inductive couple coils. The ME device can produce 21.3 mW/mm^3^ and 31.3 µW·mm^−3^ under the standards of the Institute of Electrical and Electronics Engineers (IEEE) and the International Commission on Non-Ionizing Radiation Protection (ICNIRP), respectively, with a resonance frequency of 99.3 kHz (Figure 17a)) [107].

A non-volatile and flexible ME SmFeO_3_/P(VDF-TrFE) nanocomposite memory device was introduced by Ahlawat et al. [108]. The nanocomposite film allowed electrically controlled magnetic switching with a ME coefficient of 45 mV·cm^−1^·Oe^−1^ at 1 kOe and 16 mV·cm^−1^·Oe^−1^ at 0 Oe. By applying positive and negative electric fields, the ME coefficient can switch states. The information stored using the ME coefficient shows the key advantage of non-destructive reading of polarization of ferroelectric random-access memories. It was also stated that the voltage for switching can be tuned by varying the magnetic phase fraction [108]. Still in memory devices, Wei et al. [109], reported a giant, stable, tunable and non-volatile converse ME effect in FeAl/PIN-PMN-PT ferromagnetic/ferroelectric heterostructures at room temperature with electrical magnetization modulation that can be used for a four-state memory, with electrical-writing and magnetic-reading. High density information storage can be obtained due to giant electrical modulation of magnetization [109].

Sadeghi et al. [110], reported a ME self-sensing actuator, fabricated by stacking with epoxy and hydrostatic pressure layers of Metglas/PMN-PT/Metglas. This device can work as an actuator and as a sensor. The maximum sensitivity of the actuator at the resonance frequency of 60.7 kHz was of about 5.2 nm·mA^−1^ for the actuator and 0.64 mV·mA^−1^ for the sensor and the operation range of the actuator was from 55 nm to 386 nm and the sensor of 58 to 272 mV [110]. The device applications are summarized in Table 2.

## 6. Challenges and Future Perspectives

This review has shown that the ME effect, despite having almost lost interest of the scientific community several times, always reappeared with greater vigor, being the technological applications discussed in this work a good example of this regeneration capacity. However, not everything is a bed of roses in this research field.

In one hand, it is essential to pursue new piezomagnetic and piezoelectric materials for achieving higher magnetostrictive and piezoelectric coefficients [111]. In this context, [112] it was shown that the introduction of CaZrO3 increased electric field–induced strain behavior and stated that, nowadays it is possible to achieve high piezoelectric coefficient d_33_ over 400 pC/N even in non-textured KNN ceramics, although there still persist some serious challenges for large-scale applications. It was also suggested the creation of a real MPB as in PZT system to solve the reasonably large temperature dependence of piezoelectric properties of KNN-based ceramics, which is full of scientific interest and technical importance. To achieve this, more studies are needed on the phase structure and phase diagram of KNN systems. Still, relatively to KNN-based ceramics, it is pointed as a challenge the fact that alkali elements at A-site in KNN are easily evaporated during sintering. To avoid that it’s important the establishment of processing techniques to guarantee reliable mass production and industry scale.

On the other hand, and although significant improvements have been made to achieve high magnetic field sensitivity in two-phase ferromagnetic/ferroelectric laminate composites, improvements are needed at the levels of: (i) the coupling agent or (ii) the complete removal of the coupling agent. Such pursue for multi-phase ME materials presenting high magnetic field sensitivity, remains a big challenge [111], being the printing technologies and the new ME concepts such as the magnetoionic (MI) effect [77] possible solutions.

With respect to applications of ME sensors in real-world environments, it is often concluded that the magnetic field sensitivity is harmed by contamination of the ME signals by external vibration noise sources and that, therefore, the decrease or even the elimination of the external noise is an essential challenge for the ME sensors [112]. The encapsulation of the materials and the use of resonant frequencies outside the contamination noise range are directions that should be followed.

After the innovative development of low-cost MEMS accelerometers with a reasonable behavior in terms of resolution, sensitivity and noise level [113], it would be interesting to increase the number of studies focusing in the engineering of such sensors in real situations, involving the damping ratio and evaluation/identification of other modal parameters, such as mode shapes.

In the IoT context those sensors should gain another feature: their easy integration and usability. The optimization of ME composite structures, such as flexible ME composites that can be integrated into wearable devices is a point of indispensable improvement, namely in fabricating more usable materials and devices (higher flexibility, optimized roughness and electronic miniaturization) [114].

Looking ahead, the integration of magnetic and piezoelectric nanoparticles, or even ME nanoparticles, offers a new world of opportunities to improve the devices that already exist, or the creation of totally new device concepts. This can be extremely interesting in the biomedical research area, where the interactions between nanomaterials and biological systems can be studied/optimized. Despite being expected that the development and the application of ME materials in biology and biomedicine will be of great interest to the scientific community [115], it is essential to study the parameters that can affect the ME effect (size and shape of individual particle and the environmental conditions), know the impact of the electrostatic and magnetostatic interactions between individual nanoparticles and to find a way to characterize the local ME effect in each nanoparticle. All of these demands are the key to a rational design and optimization of ME nanoparticles for this type of applications.

Recent studies have been devoted to the improvement of point-of-care devices for biomedical diagnostics, such as lab-on-a-chip systems where magnetic nanoparticles can take different functions [116] allowing production of magnetic markers with affinities to conjugate ligands for cells, proteins, or nucleic acids, among others. Bio-friendly ME nanoparticles can detect the magnetically labelled targets, allowing an improvement in detection efficiency. Spatial resolution, relative technical simplicity and tailored biocompatibility are some of the advantages of this technology. Still in the biomedical field, ME composites will also allow the mapping of magnetic fields from smaller magnetic sources (brain) and increase the sensitivity of microfluidic analysis in the context of experiments with biological samples [112].

This time travel through materials, configurations, ME coefficients and applications evidenced that an important progress has been made in understanding chemistry/structure /property relationships of the ME coupling and that ME materials are prepared to promote noteworthy breakthroughs in applications within the IoT context. In order to become a reality, issues related to accuracy, usability, scalability, dynamic production, and connectivity must be systematically addressed.

## Figures and Tables

**Table 1:** Magnetoelectric coefficient values of polyurethane based composite films [76].

*α_p_* (C/m^2^·Oe)	PU 2%Ni	PU 2%Fe_3_O_4_	PU 2%TeD
*f*_1_ = 120 Hz	6.2 × 10^−11^	1.2 × 10^−10^	4 × 10^−11^
*f*_2_ = 1 kHz	5.3 × 10^−10^	6.15 × 10^−10^	2.2 × 10^−10^

**Table 2:** IoT ME device applications.

ME Coupling	Device Type	Ref
Direct coupling	Nanogenerator	[98]
	Energy harvesting multi-cantilever beam	[99]
	For energy harvesting in wireless sensing	[100]
	Magnetic proximity sensor	[101]
	Magnetic particle mapping	[102]
	Self-biased ME cantilever sensor	[103]
	Wireless power receiver	[107]
	Self-sensing actuator	[110]
Converse coupling	Magnetic field sensor	[104]
	Low frequency transmitter	[105]
	Antenna array	[106]
	Flexible memory	[108]
	Four-state memory	[109]
